# Anti-proliferative activities of finasteride in benign prostate epithelial cells require stromal fibroblasts and *c-Jun* gene

**DOI:** 10.1371/journal.pone.0172233

**Published:** 2017-02-14

**Authors:** Kai Wang, Song Jin, Dongdong Fan, Mingshuai Wang, Nianzeng Xing, Yinong Niu

**Affiliations:** 1 Department of Urology, Beijing Chaoyang Hospital, Capital Medical University, Beijing, China; 2 Department of Urology, Weifang Hospital of Traditional Chinese Medicine, Weifang, China; University of Michigan, UNITED STATES

## Abstract

This study aimed to identify the role of mouse fibroblast-mediated *c-Jun* and IGF-1 signaling in the therapeutic effect of finasteride on benign prostatic epithelial cells. BPH-1 cells, alone or with fibroblasts (*c-Jun*^+/+^ or *c-Jun*^-/-^), were implanted subcutaneously in male nude mice who were then treated with finasteride. The degrees of cell proliferation, apoptosis, and sizes of the xenografts were determined. BPH-1 cells were grown alone or co-cultured with mouse fibroblasts in the presence of finasteride and the level of IGF-1 secreted into the medium by the fibroblasts was determined. The proliferation-associated signaling pathway in BPH-1 cells was also evaluated. Fibroblasts and *c-Jun* promoted xenograft growth, stimulated Ki-67 expression, and inhibited BPH-1 apoptosis. Finasteride did not induce the shrinkage of xenografts in the combined-grafted groups despite repressing Ki-67 expression and inducing cell apoptosis. The addition of *c-Jun*^-/-^ fibroblasts did not promote xenograft growth. In the absence of *c-Jun* and fibroblasts, finasteride did not alter xenograft growth, Ki-67 expression, or cell apoptosis. The *in vitro* results demonstrated that when BPH-1 cells were grown in monoculture, treatment with finasteride did not induce cell death and stimulated the expression of pro-proliferative signaling molecules, while in the presence of fibroblasts containing *c-Jun*, finasteride treatment repressed epithelial cell proliferation, the level of IGF-1 in the medium, and the activation of downstream pro-proliferative signaling pathways. Taken together, our results suggest that fibroblasts, *c-Jun*, and IGF-1 play key roles in mediating stromal-epithelial interactions that are required for the therapeutic effects of finasteride in benign prostate epithelial cells.

## Introduction

Dihydrotestosterone (DHT) is the most prevalent and potent form of androgen in various human organs and tissues. 5-alpha reductase (5-AR) isozymes including 5-AR1, 5-AR2 and 5-AR3, converts testosterone to DHT, which play critical roles in the normal development of the human prostate and in the pathogenesis and progression of benign prostatic hyperplasia (BPH) and prostate cancer.

5-AR inhibitors (5-ARIs), such as finasteride and dutasteride, are currently used for the prevention and treatment of these conditions[1]. Finasteride, a 4-azasteroid analogue of testosterone, functions by acting as a potent) and specific competitive inhibitor of 5-AR2. In humans, finasteride suppresses DHT by 70.8% after 24 weeks[2]. Finasteride is therapeutically effective for BPH patients because 5-AR2 is the predominant form of the enzyme in BPH[3–5].

Traditionally androgen-androgen receptor (AR) is generally accepted as the pathway which is directly associated with the pathogenesis and progression of BPH. In recent years, signaling pathways other than the androgen-AR pathway, such as the IGF-1 paracrine pathway, have garnered increasing attention because of their roles in mediating stromal and epithelial cell interactions. For example, stromally expressed c-JUN has been shown to activate pro-proliferative signaling in prostate epithelial cells by regulating paracrine IGF-1 secreted by stromal cells[6, 7].

To verify whether finasteride represses epithelial proliferation through inhibition function of *c-Jun*, therefore, repressing paracrine IGF-1, a xenograft nude mouse model was established using benign human prostatic epithelial cells (BPH-1) and mouse fibroblasts. This study aimed to identify the role of *c-Jun* and IGF-1 signaling in the therapeutic effect of finasteride on benign prostatic epithelial cells. Furthermore, the downstream molecular pathways associated with proliferation in epithelial cells were investigated.

## Materials and methods

### 2.1 Animals

Male athymic Balb/c-nu mice, 4–6 weeks old, were housed in laminar flow racks and provided with sterilized food and drinking water. Sterilized gloves, clean gowns, facemasks, and caps were used when handling the animals. All animals were purchased from and used for experiments at the Cancer Institute, Chinese Academy of Medical Sciences. All of the experiments were approved by the Institutional Animal Care and Use Committee of the Cancer Institute, Chinese Academy of Medical Sciences, and were performed at the Cancer Institute in accordance with the Principles of Laboratory Animal Care, NIH publication Vol 25, revised 1996.

### 2.2 Establishment of the grafted mouse model

Eight mice in each experimental group were subcutaneously injected with 1 × 10^7^ BPH-1 cells or mixtures of BPH-1 and *c-Jun* wild-type (*c-Jun*^*+/+*^) fibroblasts or *c-Jun* knockout (*c-Jun*^*-/-*^) fibroblasts in a ratio of 1:2.5 using a 25-gauge needle. The cells were suspended in 250 μl of phosphate buffered saline (PBS) and mixed with 250 μl of Matrigel (Becton Dickinson Labware, Bedford, MA, USA). Once the xenografts grew to approximately 100 mm^3^, the animals were treated with finasteride (intragastric feeding, 0.1 mg kg^-1^ d^-1^) or with the same amount of corn oil as a control for 5 weeks, and the length and width of the xenografts were measured twice a week using vernier calipers. The volumes of the xenografts were calculated by the modified ellipsoid formula: length × width^2^ × 0.52. After 5 weeks, xenograts’ growth had a steady tendency, in order to avoid the enormous xenograft’s effect to mice, the mice were anesthetized by injecting 10% chloral hydrate and followed by cervical dislocation in order to minimize their suffering. Then xenografts were removed, fixed by formalin and embedded by paraffin.

### 2.3 Immunohistochemistry staining of Ki-67, CK, and vimentin

The graft tissues were evaluated to assess cell proliferation using an anti-Ki-67 antibody and anti-CK and vimentin antibodies were used to determine the origin of the cells in the tissue, that is, CK positive epithelial cells or vimentin positive stromal cells. Immunohistochemistry was performed as previously described[6]. Briefly, formalin-fixed, paraffin-embedded tissue blocks were cut into 5-μm sections and mounted on positively charged slides. Tissue sections were deparaffinized with xylene and rehydrated with graded alcohol solutions then incubated in citrate buffer (pH 6.0) at 210°C for 10 min and at 160°C for 8 min in a pressure cooker. After being incubated in 3.0% hydrogen peroxide for 10 min and washed with PBS, tissue sections were immersed in a solution of rabbit anti-human Ki-67 monoclonal antibody with a dilution ratio of 1:100 (Maixin-Bio, Fuzhou, China), rabbit anti-P-CK/cytokeratin AE1 + AE3 (Bioss, Beijing, China), or rabbit anti-vimentin (Bioss, Beijing, China) for 1 h at 37°C. Tissue sections were exposed to a secondary antibody (MaxVisionTM HRP-polymer anti-rabbit IHC kit, Maixin-Bio, Fuzhou, China) for 15 min at room temperature. Finally, the sections were incubated in DAB chromogen and then counterstained with hematoxylin. A BPH specimen which was known to express strong Ki67, CK and vimentin proteins as positive control and the same specimen without adding primary antibody against Ki67, CK and vimentin as negative control although the controls were not shown in the manuscript. The ratio of Ki-67-positive cells was defined as the percentage of Ki67-positive cells in 2000 BPH-1 cells which was randomly selected under microscope at 200 × magnification.

### 2.4 Tunel

The apoptotic cancer cells in the xenografts were identified using the ApopTag® Peroxidase *In Situ* Apoptosis Detection Kit (Millipore Corporation, Billerica, MA, USA) in accordance with the manufacturer’s instructions. The working solution of antibody is included in the kit. The apoptotic index was defined as the percentage of apoptotic cells in 2000 BPH-1 cells which was randomly selected under microscope at 200 × magnification.

### 2.5 Cells and cell culture conditions

Initiated benign prostate epithelial cells (BPH-1), obtained from the Cell Resource Center, the Institute of Basic Medical Sciences, Chinese Academy of Medical Sciences, were cultured in RPMI 1640 medium (Gibco, Rockville, MD, USA) supplemented with 2 mmol L^-1^ L-glutamine, 10% fetal bovine serum (FBS) (Gibco, Melbourne, Australia), and 1% penicillin-streptomycin (Hyclone, Logan, Utah, USA) at 37°C with 5% CO_2_. *c-Jun*^*+/+*^ and *c-Jun*^*−/−*^ mouse embryonic fibroblasts were cultured in Dulbecco’s modified Eagle’s medium (DMEM) supplemented with 2 mmol L^-1^ L-glutamine, 10% FBS, and 1% penicillin-streptomycin at 37°C with 5% CO_2_. The co-culture experiments were performed as described previously[6]. Briefly, BPH-1 cells were cultured in 0.4 μm pore size permeable membrane transwell inserts (Corning Inc., Corning, NY, USA), and mouse fibroblasts were cultured in 6-well plates. When 50% confluent, BPH-1 cells were starved in FBS-free DMEM for 24 h, then moved to the 6-well plate with 80% confluent fibroblasts. The stromal-epithelial co-cultures were maintained in 1% FBS DMEM medium containing 100 μmol L^−1^ finasteride (LKT Laboratories, Inc., St. Paul, MN, USA) previously prepared in DMSO, and the medium was replaced every 24 h for 72 h.

### 2.6 Cell extracts and western blot analysis

The cells were harvested to obtain total cell lysates with M-PER Mammalian Protein Extraction Reagent (Pierce Biotechnology, Rockford, IL, USA) containing a mixture of protease inhibitors (Halt Protease Inhibitor Cocktail Kit, Pierce Biotechnology, Rockford, IL, USA) according to the instructions. The protein concentration was determined by the BCA protein assay reagent (Pierce, Rockford, IL, USA). The western blot analysis was performed as previously described[6]. The antibodies used for the western blot analysis included antibodies against: AKT, phospho-AKT (Ser473), extracellular signal-regulated kinase (ERK), phospho-ERK (Thr202/ Tyr204), cyclin D1, and cyclin D3, which were all purchased from Cell Signaling (Boston, MA, USA). The horseradish peroxidase-conjugated secondary antibodies (goat anti-mouse and goat anti-rabbit) were obtained from Santa Cruz Biotechnology Inc. (Santa Cruz, CA, USA), and the GAPDH antibody was purchased from Abcam, Inc. (Cambridge, MA, USA).

### 2.7 Cell proliferation assay

Cell proliferation was assessed by the MTS assay in accordance with the manufacturer’s instructions (Cell Titer 96^®^ Aqueous One Solution Cell Proliferation Assay, Promega; Madison, WI, USA). Briefly, pipet 20μl of Cell Titer 96® AQueous One Solution Reagent containing a novel tetrazolium compound into each well of the 96-well assay plate containing the samples in 100μl of culture medium, incubate the plate at 37°C for 2 hours in a humidified, 5% CO2 atmosphere, the tetrazolium compound is bioreduced by metabolically active cells into a colored formazan product that is soluble in tissue culture medium, record the absorbance at 490nm using a 96-well plate reader. The normalized cell proliferation is calculated as the percentage of measured absorbance of cell viability to that of control group (100%).

### 2.8 Enzyme-linked immunosorbent assay

An IGF-1-specific enzyme-linked immunosorbent assay (ELISA) kit (R&D Systems Inc., Minneapolis, MN, USA) was used to measure the IGF-1 levels in media obtained from a 72-h co-culture system. The assays were performed according to the manufacturer’s instructions, and the luminescence was measured at 450 nm using a plate reader.

### 2.9 Semiquantitative reverse transcriptase-polymerase chain reaction analysis

RNA isolation was performed using an RNeasy mini kit (Qiagen Sciences, MD, USA). For each 25-μl semiquantitative reverse transcriptase-polymerase chain (RT-PCR) reaction, 0.5 μg of RNA was used and cDNA synthesis was achieved by 50°C for 30 min, predenaturation at 94°C for 5 min, and then PCR amplification was performed for 30 cycles: denature at 94°C for 50 sec, anneal at 56°C for 50 sec, and extend at 72°C for 1 min, final extension at 72°C for 10 min. In each reaction, GAPDH was used as an internal control. The primers used for PCR were as follows: BPH-1 AR, 5’-CCGCTGACCTTAAAGACATCC-3’ (forward) and 5’- CGACACT GCCTTACACAACTC’ (reverse); BPH-1 5-AR1, 5’- GAAACTTGCCAACCTTCG TG -3’ (forward) and 5’- CTTACTCCGTATGAACCACCA -3’ (reverse); BPH-1 5-AR2, 5’- CTCTTCTGCCTACATTACTTCCA -3’ (forward) and 5’- CACCCAA GCTAAACCGTATGTC -3’ (reverse); BPH-1 GAPDH, 5’-TGATGACATCAAGA AGGTGGTGAAG-3’ (forward) and 5’-TCCTTGGAGGCCATGTGGGCCAT-3’ (reverse). Mouse fibroblasts AR, 5’- ACAACAACCAACCAGATTCC -3’ (forward) and 5’- CAAATACCATCAGTCCCATCC-3’ (reverse); Mouse fibroblasts 5-AR1, 5’- CCCTGCTGTTCACCTTTGTC -3’ (forward) and 5’- TATTTATCACCATGCC CACTAACC -3’ (reverse); Mouse fibroblasts 5-AR2, 5’- CTGAAAGCCACTGCCT TCTG -3’ (forward) and 5’- CCCATCCATTCAATAATCTCGCC -3’ (reverse); Mouse fibroblasts GAPDH, 5’- AGGTCGGTGTGAACGGATTTG -3’ (forward) and 5’- TGTAGACCATGTAGTTGAGGTCA? -3’ (reverse); IGF-1, 5′-ATGTCGT CTTCACACCT-3′ (forward) and 5′-ACTTGTGTTCTTCAAA TGTACTTCC-3′ (reverse). The PCR products were electrophoresed in 1% agarose gels, stained with ethidium bromide, and then photographed.

### 2.10 Statistical methods

Relevance Among the growth curves of grafted tumors was analyzed using repeated measures ANOVA test, Associations between c-Jun, fibroblasts, finasteride and tumor growth were evaluated by the ANOVA and LSD test using SPSS version 16 (SPSS Inc., Chicago, IL, USA).

## Results

### 3.1 AR and 5-AR expression in BPH-1 and mouse fibroblasts

**Fig 1:**
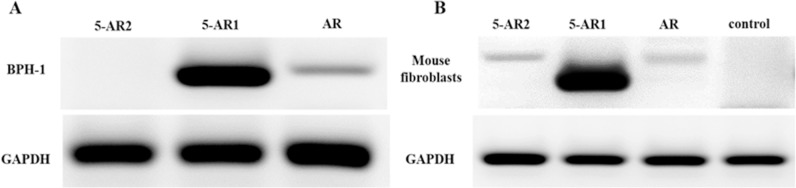
Expression of AR, 5-AR1 and 5-AR2 in mouse fibroblast and BPH-1 cells. (A) mRNA transcription of AR and 5-AR in BPH-1 cells. Low level of AR mRNA, high level of 5-AR1 mRNA and negative 5-AR2 mRNA were detected in BPH-1 cells. (B) mRNA transcription of AR and 5-AR in mouse fibroblasts. A small quantity of AR mRNA and 5-AR2 mRNA, high level of 5-AR1 mRNA were found in mouse fibroblasts.

The presence of AR and 5-AR in BPH-1 cells and mouse fibroblasts were checked by RT-PCR (Fig 1). Low level of AR mRNA and high level of 5-AR1 mRNA were detected, while 5-AR2 mRNA was negative in BPH-1 cells (Fig 1A). On the other hand, mouse fibroblasts express a small quantity of AR mRNA and 5-AR2 mRNA, and high levels of 5-AR1 mRNA (Fig 1B).

### 3.2 Fibroblasts are critical for finasteride-induced BPH-1 cell death in vitro

**Fig 2:**
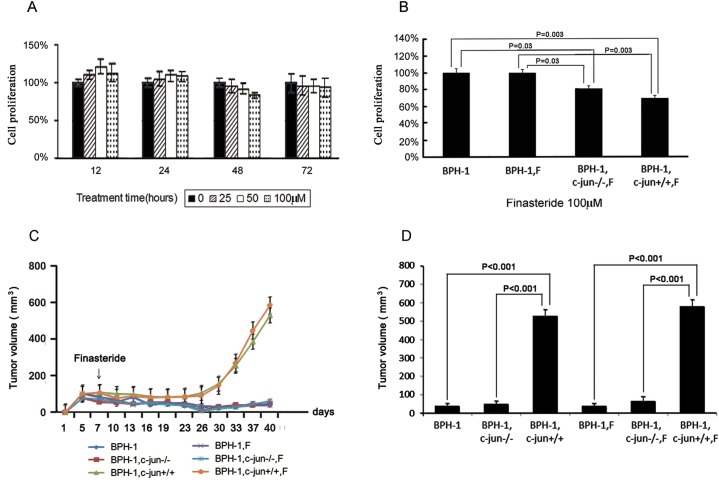
Cell proliferation and apoptosis in vitro and in xenografts of BPH-1-fibroblast-combined-grafted model. (A) Cell proliferation of BPH-1 cells in mono-culture. High concentrations of finasteride did not inhibit cell proliferation in mono-culture. (B) Cell proliferation of BPH-1 cells co-cultured with wild-type fibroblasts or *c-Jun*^-/-^ fibroblasts. In the presence of fibroblasts, finasteride repressed cell proliferation by 30% (p = 0.003). (C&D) Growth curve of xenografts in the BPH-1-fibroblast-combined-grafted model. Compared with the BPH-1 mono-grafted group, wild-type fibroblasts (*c-Jun*^+/+^) promoted xenograft growth whereas *c-Jun*^-/-^ fibroblasts did not stimulate xenograft growth. Finasteride did not have a significant impact on xenograft growth in the presence or absence of fibroblasts in limited 5-week experimental period.

In mono-culture, the treatment of BPH-1 cells with high concentrations of finasteride failed to induce cell death (Fig 2A); however, when BPH-1 cells were co-cultured with mouse fibroblasts, finasteride treatment induced BPH-1 cell death by 30% (*P* = 0.003, Fig 2B).

### 3.3 Fibroblasts stimulate xenograft growth in a BPH-1- fibroblast grafted mouse model in the absence of finasteride

**Fig 3:**
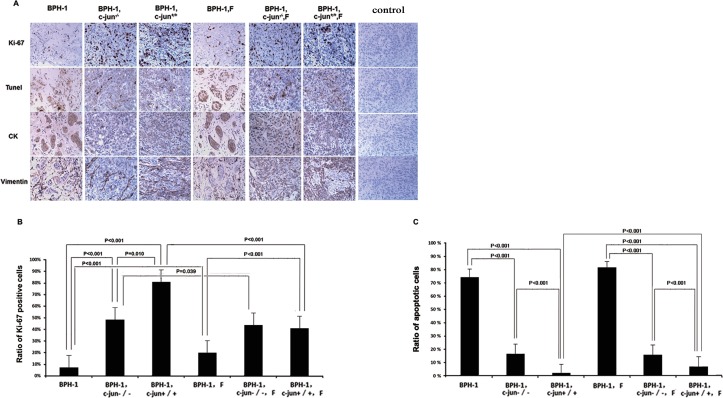
Cell proliferation and apoptosis in xenograft tissues. (A) Immunostaining of Ki-67, CK, vimentin and TUNEL in xenografted tissues. (B) Fibroblasts and *c-Jun* promoted the expression of Ki-67 in BPH-1 cells. Finasteride decreased the ratio of Ki-67-positive cells in epithelial cells in BPH-1-fibroblasts (*c-Jun*^+/+^) combined-grafted group. (C) Fibroblasts and *c-Jun* repressed the ratio of apoptotic BPH-1 cells, whereas finasteride promoted cell apoptosis in the presence of fibroblasts.

In the combined-grafted model, mouse fibroblasts promoted xenograft growth over that observed in the mono-grafted groups (526.2 mm^3^ versus 38.2 mm^3^, *P* < 0.001, Fig 2C and 2D), and the degrees of cell proliferation and apoptosis presented reasonable concordance. Compared with the control group, the presence of fibroblasts promoted Ki-67 expression (8.12% versus 81.05%, *P* < 0.001) and decreased apoptosis (74.42% versus 2.18%, *P* < 0.001) in BPH-1 cell populations (Fig 3).

### 3.4 Finasteride represses BPH-1 cell proliferation and induces cell apoptosis in a BPH-1-fibroblast grafted mouse model

Finasteride treatment did not impact the size of BPH-1 xenografts in the presence of fibroblasts (526.2 mm^3^ versus 579.5 mm^3^, *P* = 1.0, Fig 2C and 2D) after 5-week treatment. However, a decreased ratio of Ki-67-positive epithelial cells (Fig 3B; 41.00% versus 81.05%, *P* < 0.001) and an increased ratio of apoptotic cells (Fig 3C, 7.35% versus 2.18%, *P* = 0.019) were observed in the combined-grafted group treated with finasteride compared with the combined-grafted group not treated with finasteride.

### 3.5 c-Jun is required for the therapeutic effects of finasteride in BPH-1 xenografts

The genetic deletion of *c-Jun* (*c-Jun*^*-/-*^) compromised the pro-proliferative effects of fibroblasts for BPH-1 xenograft growth (49.1 mm^3^ versus 526.2 mm^3^, *P* < 0.001). Moreover, finasteride does not exert an anti-proliferative effect on BPH-1 xenografts in the presence of *c-Jun*^-/-^ fibroblasts (49.1 mm^3^ versus 63.2 mm^3^, *P* = 0.835). (Fig 2C and 2D).

In the presence of *c-Jun*^-/-^ fibroblasts, the ratio of Ki-67 positive BPH-1 cells was higher than observed in the BPH-1 mono-grafted group (49.50% versus 8.12%, *P* < 0.001), but was lower than that found in the presence of wild-type fibroblasts (49.50% versus 81.05%, *P* < 0.001). Knocking out *c-Jun* compromised the proliferation-repressing effect of finasteride, which only slightly reduced Ki-67 expression in the presence of *c-Jun*^-/-^ fibroblasts (49.50% versus 44.80%, *P* = 0.039). (Fig 3C and 3D)

The presence of *c-Jun* was shown to play a role in mediating cell apoptosis, the percentage of apoptotic epithelial cells was higher in co-cultures with *c-Jun*^-/-^ than that observed in co-cultures with wild-type fibroblasts (16.40% versus 2.18%, *P* < 0.001). Finasteride did not affect BPH-1 cell apoptosis in the presence of *c-Jun*^-/-^ fibroblasts (16.40% versus 16.80%, *P* = 0.752). (Fig 2C).

### 3.6 Finasteride inhibits IGF-1 synthesis in fibroblasts co-cultured with BPH-1 cells

**Fig 4:**
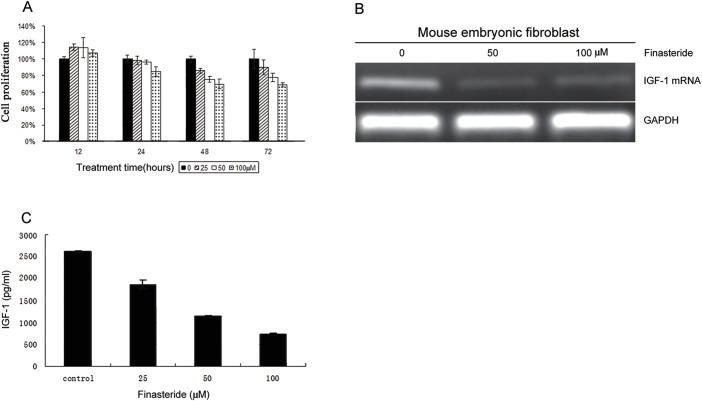
The therapeutic effect of finasteride on fibroblasts. (A) Finasteride induced cell death of fibroblasts. (B) Finasteride repressed the transcription of IGF-1 mRNA in fibroblasts in epithelia-fibroblasts co-culture system. (C) The level of IGF-1 protein in the medium of co-culture system was decreased in the presence of finasteride.

Finasteride treatment induced cell death of fibroblasts (Fig 4A) and decreased the IGF-1 mRNA levels in fibroblasts that are maintained in a co-culture system of epithelial cell and fibroblast (Fig 4B). In addition, the level of IGF-1 protein detected in the medium of co-cultured cells was significantly lower following treatment with finasteride (Fig 4C).

### 3.7 Finasteride reduces the expression of p-AKT and p-ERK1/2 in BPH-1 cells co-cultured with fibroblasts in vitro

**Fig 5:**
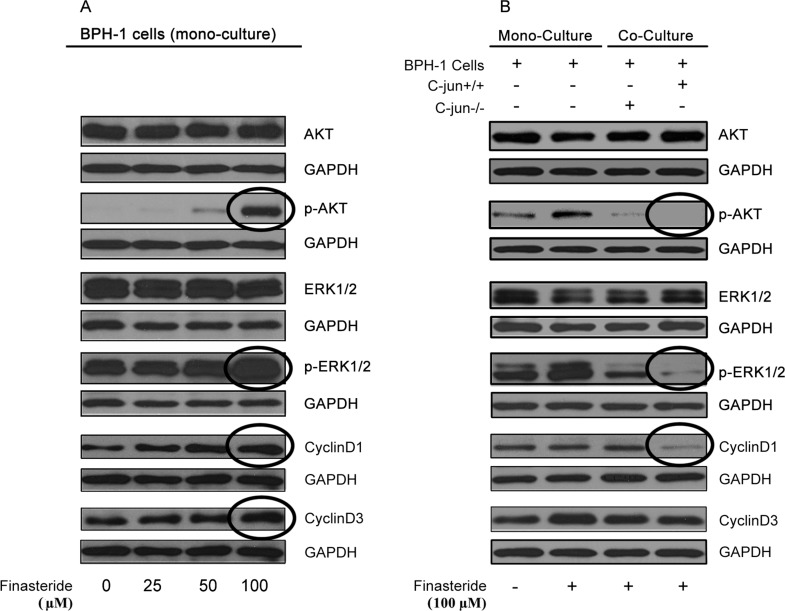
The effect of finasteride on proliferation-associated signaling pathway in BPH-1 in mono-culture or co-culture with fibroblasts. (A) Finasteride stimulated the expression of p-AKT, p-ERK1/2, cyclin D1 and cyclin D3 in a concentration-dependent manner in BPH-1 mono-culture. (B) Finasteride repressed the expression of p-AKT, p-ERK1/2 and cyclin D1 in BPH-1 cells when co-cultured with fibroblasts. The inhibiting effect of finasteride on downstream proliferation-associated signaling pathway was compromised by *c-Jun* knockout in fibroblasts.

Through a western blot analysis, we showed that finasteride stimulates the expression of p-AKT, p-ERK1/2, and downstream cyclin D1 and cyclin D3 signaling in BPH-1 cells maintained in mono-culture. In contrast, in BPH-1 cells co-cultured with wild-type fibroblasts, finasteride repressed the expression of p-AKT (1.21 versus 0.33, *P* < 0.001), p-ERK1/2 (1.16 versus 0.21, *P* < 0.001), and cyclin D1 (0.82 versus 0.16, *P* < 0.001). In BPH-1 cells co-cultured with *c-Jun*^-/-^ fibroblasts and exposed to finasteride, higher expression levels of p-AKT (0.43 versus 0.33, *P* = 0.06) and p-ERK1/2 (0.21 versus 0.69, *P* <0.001) were observed (Fig 5). Therefore, *c-Jun* was shown to play a critical role in mediating the therapeutic effects of finasteride.

## Discussion

In this study, we demonstrated that finasteride does not induce cell death in benign prostate epithelial cells that are maintained in mono-culture, and that the anti-proliferation activity of finasteride is dependent on the co-culture of benign prostate epithelial cells with wild fibroblasts containing intact *c-Jun*. Finasteride cannot exert any direct impact on BPH-1 cells because they do not express 5-AR2. On the other hand, mouse fibroblasts express 5-AR2, and both BPH-1 cells and fibroblasts express a small quantity of AR. Based on these findings, we hypothesize that finasteride induces epithelial cell death through crosstalk between the epithelial and stromal compartments, which is associated with either androgen-AR signaling or other non-androgen-AR pathways.

The prostate is a tubuloalveolar gland composed of epithelial tissues that are embedded within stromal components. The main cell types in the stroma include fibroblasts, myofibroblasts, and smooth muscle cells. Stromal cells secrete growth factors[8], produce extra-cellular matrix (ECM), and express AR, estrogen receptors, adrenergic receptors, and 5-AR[9–11]. The stromal compartment plays a critical role in the development of BPH and prostate cancer[12–14]. As men age, their serum sex hormone profiles change, which may be critical for the induction of BPH by the stroma compartment. A study conducted by John McNeal demonstrated that the neo-formation of the ductal-acinar architecture during BPH pathogenesis is because of the reactivation of embryonic inductive activity by hyperplastic stroma[15], reinforcing the hypothesis that the stromal microenvironment is important for prostate re-growth and for the therapeutic effects of finasteride on BPH.

We verified that stromal fibroblasts promote xenograft growth in grafted mouse models. The balance between the proliferation and apoptosis of epithelial cells was altered by the presence of fibroblasts, and fibroblasts were shown to promote xenograft growth[16, 17]. Identifying the signal transduction pathways between epithelia and the surrounding stroma will improve our understanding of the abnormal biology observed in prostatic diseases. Factors other than androgen-AR interactions are involved in prostatic diseases. Multiple growth factors, chemokines and their corresponding receptors, and subsequent downstream signaling pathways have been shown to play a role in the development and progression of prostate diseases via autocrine or paracrine signaling within the stromal microenvironment. Although a study conducted by Li et al. [6] suggested that fibroblasts promote prostatic epithelial proliferation through the IGF-1 paracrine pathway, it was unclear whether finasteride acts through the same pathway. In our experiments, finasteride inhibited Ki-67 expression and induced cell apoptosis in epithelial cells when co-cultured with fibroblasts, though no tumor shrinkage was observed in the limited experimental period compared with the long therapeutic time of months or years for men taking finasteride. Finasteride may reduce the prostate volume by repressing proliferation and inducing apoptosis of epithelial cells through paracrine crosstalk with stromal cells over longer time periods.

5-AR is an important enzyme in BPH. Both 5-AR1 and 5-AR2 are present in epithelial and stromal cells as we found in human BPH tissues, and 5-AR2 is the predominant isozyme in stromal cells[4, 18]. Importantly, fibroblast-secreted soluble factors have been shown to induce the transcription of 5-AR2 mRNA in long-term primary cultures of prostate epithelial cells that can no longer transcribe 5-AR2 mRNA in the absence of fibroblasts[19]. Fibroblast-secreted factors are very important for the transcription and translation of 5-AR2 through stroma-epithelia crosstalk and for the therapeutic effects of finasteride in BPH.

The JUN family of proteins are critical transcription factors that act as co-activators of AR or form activator protein 1 with FOS to regulate the transcription of androgen-regulating genes. Chen[20] reported that c-JUN is an AR co-activator that stimulates AR transactivation by mediating receptor dimerization and subsequent DNA binding. In our study, we observed the pro-proliferative effects of *c-Jun*-expressing and AR-positive mouse fibroblasts on epithelial cells, which is consistent with the results reported by Li that stromally expressed protein of c-JUN promotes prostatic epithelial proliferation through the IGF-1 paracrine pathway[6]. We also found that knockout of *c-Jun* compromised the pro-proliferative function of fibroblasts and attenuated the repressive effects of finasteride on proliferation of epithelial cells to a much lower level. Taken together, these results suggest that *c-Jun* plays a critical role in the pro-proliferative function of fibroblasts and in the anti-proliferative activity of finasteride on epithelial cells.

Our study showed that finasteride inhibits the synthesis and secretion of IGF-1 by fibroblasts, similar to the results in *c-Jun*-depleted fibroblasts reported by Li[6]. We postulate that finasteride inhibits the expression of IGF-1 by repressing the activity of c-JUN. However, further experiments are required to investigate whether c-JUN promotes the expression of IGF-1 by binding to the promoter region as a transcription factor, and to elucidate the mechanism through which finasteride inhibits the function of c-JUN.

We also assessed the downstream signaling pathways that are closely related to cell proliferation. Although finasteride promotes the expression of p-AKT and p-ERK1/2 in epithelial cells maintained in mono-culture, finasteride represses p-AKT and p-ERK1/2 expression in epithelial cells that are co-cultured with fibroblasts. Finasteride inhibits pro-proliferative signals in epithelial cells through its impact on stroma-epithelia crosstalk. The growth of epithelial cells with *c-Jun*^-/-^ fibroblasts compromised the repressive effect of finasteride on the levels of p-AKT and p-ERK1/2, underlining the importance of *c-Jun* in epithelia-fibroblast interactions and in the therapeutic function of finasteride.

Here, we need to mention some limitations in this investigation. First, although fibroblasts have been reported to induce the proliferation of prostate epithelial cells[6, 13], the data in our article are not perfect to explain the inducing effect of fibroblasts for epithelial cells, because in recombinant‑grafted group, we implanted fibroblasts in addition to epithelial cells compared with the epithelial cells in mono-grafted group, However, the pro-proliferative function of fibroblasts was still observed characterized by increased Ki-67 expression and repressed apoptosis in the epithelial cells. In this article, we focused on the role of fibroblasts and *c-Jun* for the therapeutic effects of finasteride for prostatic epithelial cells, as above discussed. Second, the duration of the animal experiments was 5 to 6 weeks, which is markedly shorter than the therapeutic time of months or years for men taking finasteride; therefore, it is hard to show the effects of finasteride fully on prostatic epithelial cells although we have already found that finasteride repressed proliferation and promoted apoptosis in prostatic epithelial cells in the presence of fibroblasts. Third, although the application of Matrigel is helpful for BPH-1 xenograft growth, particularly in the mono-grafted group, this may represent a confounding factor when evaluating the role of fibroblasts on the effect of finasteride on prostatic epithelial cells. We applied Matrigel to all of the groups and aimed to eliminate this confounding impact by maintaining the same volume and the same concentration of Matrigel.

## Conclusions

Fibroblasts and *c-Jun* promote xenograft growth. In the absence of fibroblasts, finasteride did not induce cell death and activated pro-proliferative molecular pathways in prostate epithelial cells. In the presence of fibroblasts, finasteride treatment was found to repress epithelial cell proliferation, increase cell apoptosis, reduced the level of IGF-1 in the medium and inhibited the activation of downstream signaling pathways. Taken together, our results suggest that fibroblasts, *c-Jun* and IGF-1 play key roles in mediating the stroma-epithelia interactions that are required for the therapeutic effects of finasteride on benign prostate epithelial cells.

## Supporting information

S1 File NC3Rs ARRIVE Guidelines Checklist (fillable). (PDF)

S2 File Data. **1. Fig 2A and Fig 4A data.** BPH-1 and fibroblasts cell proliferation after finasteride treatment. **2. Fig 2B data.** Cell viability of BPH-1 in co-culture with fibroblasts upon 100uM finasteride treatment. **3. Fig 2C & 2D data.** Animal data. **4. Fig 3B & 3D data.** Results of Ki67 and Tunel about xenograft immunohistochemistry. **5. Fig 4C data.** IGF-1 concentration in co-culture system upon treatment of finasteride.
(RAR)
